# Comparing Gait Trials with Greedy Template Matching

**DOI:** 10.3390/s19143089

**Published:** 2019-07-12

**Authors:** Aliénor Vienne-Jumeau, Laurent Oudre, Albane Moreau, Flavien Quijoux, Pierre-Paul Vidal, Damien Ricard

**Affiliations:** 1 COGNAC-G (UMR 8257), CNRS Service de Santé des Armées University Paris Descartes, 75006 Paris, France; 2 L2TI, University Paris 13, 93430 Villetaneuse, France; 3 CMLA (UMR 8536), CNRS ENS Paris-Saclay, 94235 Cachan, France; 4 ORPEA Group, 92813 Puteaux, France; 5 Hangzhou Dianzi University, 310005 Hangzhou, China; 6 Service de Neurologie, Hôpital d’Instruction des Armées Percy, Service de Santé des Armées, 92190 Clamart, France; 7 Ecole du Val-de-Grâce, Ecole de Santé des Armées, 75005 Paris, France

**Keywords:** inertial measurement units, gait analysis, biomedical signal processing, pattern recognition, step detection, physiological signals

## Abstract

Gait assessment and quantification have received an increased interest in recent years. Embedded technologies and low-cost sensors can be used for the longitudinal follow-up of various populations (neurological diseases, elderly, etc.). However, the comparison of two gait trials remains a tricky question as standard gait features may prove to be insufficient in some cases. This article describes a new algorithm for comparing two gait trials recorded with inertial measurement units (IMUs). This algorithm uses a library of step templates extracted from one trial and attempts to detect similar steps in the second trial through a greedy template matching approach. The output of our method is a similarity index (SId) comprised between 0 and 1 that reflects the similarity between the patterns observed in both trials. Results on healthy and multiple sclerosis subjects show that this new comparison tool can be used for both inter-individual comparison and longitudinal follow-up.

## 1. Introduction

Gait semiology is of major importance in neurological practice, as abnormalities are associated with high comorbidities. The quantification of gait using inertial measurement units (IMUs) has become a democratic method for the follow-up of subjects with locomotion alterations in healthcare. The use of such embedded technologies has already shown its usefulness in the detection of postural strategies during walking [1], partitioning gait during the stance phase [2] or motor supplementation for switch-activated simulators [3]. However, these clinical applications require the detection of steps within the IMU signals. Spatio-temporal gait parameters can also be extracted for the healthy or disabled and stored in databases that enable a longitudinal follow-up of patients with gait disorders due to ageing [4], orthopedic or rheumatic diseases [4,5,6] or neurological alterations [4,7,8,9]. It has proven useful to help clinicians refine the description of individual gait disorder and strengthen their insights into the patients’ movements and compensation patterns. This quantification of characteristics related to altered gait using signals from IMUs that are collected inside databases allows inter-individual comparisons to assess the distance of the patients’ gait from a control group [10] or intra-individual comparisons for longitudinal follow-up [11,12]. Common gait features most often rely on basic statistics such as averages or standard deviations over a whole exercise [13,14]. On the one hand, they provide useful and interpretable information for the clinician. On the other hand, they have not proven sensitive enough for detecting subtle changes in several pathologies [4,15,16]. Besides, they display high inter-session variability for diseases that present with day-to-day changes [17]. To assure robustness of these parameters, it is usually necessary to increase the number of steps within a trial [18,19,20]. However, repeated measures or treadmill exercises are incompatible with common clinical practice in patients with limited walking perimeter, which is frequent in neurological practice. In order to obtain a more integrative perspective, some authors resort to global indexes, which are composed of several parameters [21,22]. These scores are promising but careful consideration should be given to their evolution inasmuch as the absence of evolution of a multicomponent score does not necessarily reflect the reproducibility of the gait pattern between two measurements [20]. Indeed, maintaining a steady value of an overall score over time may mask gait adaptations. For instance, gait velocity may be maintained despite a decreased step length if the cadence increases concomitantly. What is more, these parameters heavily rely on accurate step detection, which is problematic in severely altered steps: Some patients may require manual painstaking and time-consuming detection [23]. It is therefore key to evaluate this detection or be exempt from it.

Progressive multiple sclerosis (pMS) is one of the disorders that benefits heavily from the use of IMUs in routine clinical practice to assess indices of the disease evolution [24,25,26]. IMUs have been applied to reliably monitor patients’ health status with regard to their risk of falling [27], their physical abilities [28,29] or the neuromotor strategies used to adapt to their disability [22,30,31,32]. However, patients suffering from severe pMS may impose high constraints both on measurements, which should be short and controlled to abstract from fatigue and day-to-day variations, and processing, which should adapt to very abnormal patterns and confounders such as false gait events triggered off by loading and unloading of walking aids.

In this study, a new metric to compare two gait trials recorded with IMUs, which we called “similarity index” (SId), is introduced. It aims at overcoming previously mentioned withdrawals of statistical methods in pathologies such as pMS. The SId is an asymmetrical metric that takes as input two gait trials and computes an index, comprised between 0 and 1, that assesses the similarity between them. It is hypothesized that such a metric provides a valid characterization of a change in gait patterns between two measurements, and can therefore be used either for inter-individual comparison or longitudinal follow-up.

First, the SId is compared between pairs of trials of increasing distance. Second, it is evaluated against more conventional features to estimate its capacity to assess changes in gait. Eventually, its ability to indicate the level of confidence of the underlying step detection is appraised.

## 2. Data, Protocol and Subjects

### 2.1. Protocol

**Figure 1:**
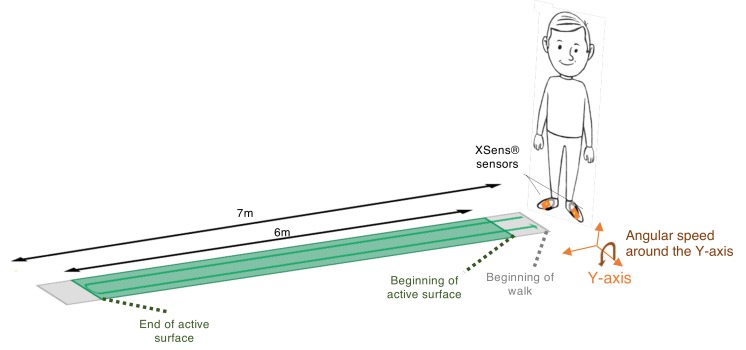
Measurement protocol. The XSens${}^{®}$ sensors (XS) inertial measurement units and the GaitRite${}^{®}$ mat (GR) are synchronized by using the PC clock connected to the inertial measurement units. The active surface (green) is covered with pressure sensors. The rest of the mat (grey) is inactive and does not detect any pressure from the subject.

Two XSens${}^{®}$ sensors (Xsens${}^{®}$ Technologies, Enschede, the Netherlands)—hereafter XS—were placed on the participant’s body (one on the dorsal part of each foot) using Velcro bands. The XS showed high reliability at heel impact for the ankle joint in the sagittal plane (inter-correlation coefficient (ICC) > 0.8) [33]. With a standard error of the mean (SEM) below 3°, between- and within-rater reliability of kinematic variables obtained from XS across joints and planes, its consistency is comparable to or better than that obtained from optoelectronic motion capture systems [34]. The GaitRite${}^{®}$ mat—hereafter GR—exhibits strong concurrent validity [35,36] and excellent reliability (with ICC > 0.8) for most temporo-spatial gait parameters in both young and older subjects [37,38,39]. The GR can be used to assess people with altered gait with good reliability even though walking ability does influence it. The ICCs for the older subjects [37] or patients with neurological diseases [40,41] can be somewhat lower but are still adequate for measuring step parameters of gait in these populations. Based on this data, GR is used in this paper as the gold standard. The data were sampled at 100 Hz for the XS and at 120 Hz for the GR. Both systems were synchronized in time by using the PC clock connected to the XS. Participants performed four walks of 12 m with a U-turn (6 m on the way in and 6 m on the way out): Two at the first visit (M0) and again two at the second visit six months later (M6). The choice of a six-month period between measurement was driven by the fact that patients from the pMS group undergo routine evaluation of their gait every six months. The protocol is schematized in Figure 1.

### 2.2. Subjects

Twenty-two patients with progressive multiple sclerosis (pMS) and ten young healthy subjects (HS) were enrolled in this longitudinal study. The characteristics of the subjects are displayed in Table 1. pMS patients were consecutively recruited from the outpatient clinic of Percy Hospital (Clamart, France) between June 2018 and September 2018. The inclusion criteria for participation in this cohort required patients to be at least 18 years old, be diagnosed with primary progressive or secondary progressive multiple sclerosis according to the 2010 International Panel criteria [42], be capable of walking 20 m with U-turn and be free of any other conditions that affect gait. HS participants were recruited from the hospital and research unit staff between June 2018 and September 2018. The inclusion criteria were: No report of falls in the past five years prior to inclusion and no disease that could affect their walk. The sex ratio was comparable between the two groups and no major differences were seen between other anthropometric characteristics. pMS patients were aged 58 (±11) years old and the HS group mean age was 26 (±2) years old. The two groups were not matched for age as one aim of this analysis was to analyze the performance of the algorithm on two opposite groups, one with highly altered steps (pMS) and one with the most normal steps. Severity of the disease was evaluated using the Expanded Disease Status Scale (EDSS) [43], which is a score of 0 to 10, ranging from normal neurological examination (0) to total impotence (9.5) or even death (10). Included participants in the pMS group had an EDSS between 3.0 and 6.5, as disabilities greater than 7.0 impede walking even a few steps. Seven out of the 22 participants had an advanced disease requiring permanent walking aid (cane(s), walker and/or human help). Two patients needed human help to perform the walking test. All subjects provided a written informed consent prior to their inclusion. The study protocol followed the principles of the Declaration of Helsinki and was approved by the Ethics Committee “Protection des Personnes Nord Ouest III” under the ID RCB: 2017-A01538-45.

## 3. Method

We now define the similarity index (SId) between two gait trials. Let us consider two gait trials:

- One train trial denoted $i_{train}$ and composed of both GR data and XS data.
- One test trial denoted $i_{test}$, only composed of XS data.

The aim of the algorithm presented in this section is to compute a similarity index ${\mathrm{SId}}_{i_{test}|i_{train}}$, comprised between 0 and 1, that will assess the proximity between trials $i_{train}$ and $i_{test}$. This metric is based upon the following question: How well can the group of steps present in trial $i_{train}$ predict those observed in trial $i_{test}$ ?

**Figure 2:**
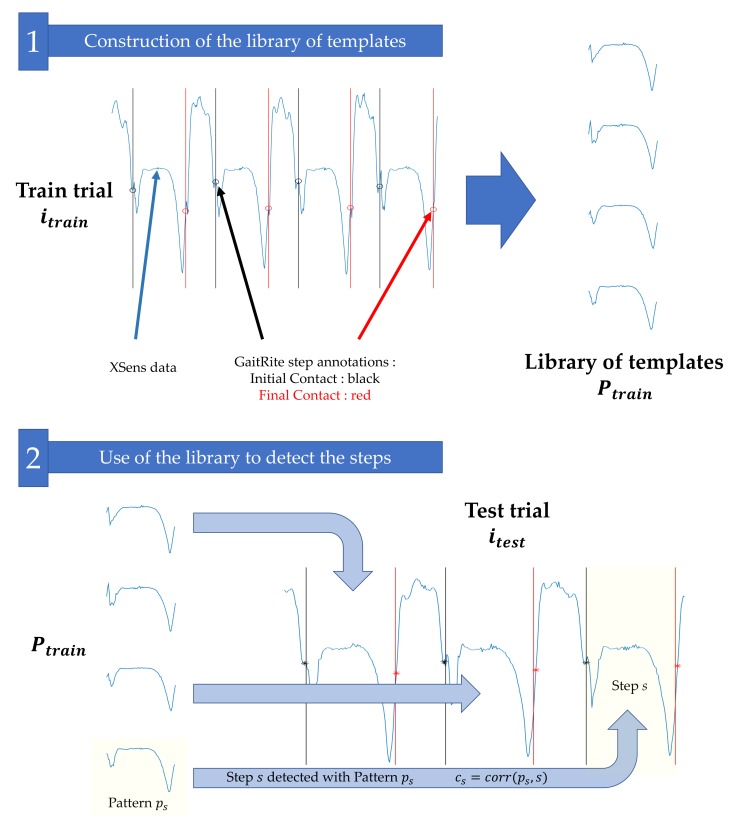
Main stages for the computation of the similarity index (SId). First, the GR and XS data from the trial $i_{train}$ are used to build a library of templates $P_{train}$. In the second stage, the library is used to detect the steps in the trial $i_{test}$, according to a greedy template-based approach inspired by [44]. Each detected step *s* is associated with one template $p_{s}$. The correlation coefficients $c_{s}$ between the steps *s* and their associated templates $p_{s}$ are then averaged to obtain the similarity index ${\mathrm{SId}}_{i_{test}|i_{train}}$.

The computation of this index is based on three main stages, detailed below and illustrated in Figure 2.

1. The GR and XS data from trial $i_{train}$ are used to build a library of templates $P_{train}$;
2. This library is used to detect the steps in trial $i_{test}$, according to a greedy template-based approach inspired by [44];
3. The Pearson coefficients between the detected steps in $i_{test}$ and the patterns in $P_{train}$ used for their detection are merged to compute the similarity index ${\mathrm{SId}}_{i_{test}|i_{train}}$.

### 3.1. Construction of the Library of Templates

Let us consider a train gait trial $i_{train}$ composed of GR and XS data. We first use the GR recordings to extract the exact timings for initial contacts (ICs) and final contacts (FCs). This process is automatically performed thanks to the GR software. Only the steps occurring while the subject is on the active surface of the instrumented mat are used; steps occurring during the U-turn are not considered. Then, we use the XS synchronized data to build the library of templates. We consider for each right/left foot XS sensor the *Y*-axis angular velocities (swing in the direction of the walk) and construct a library of templates by extracting the steps in the XS signals. More precisely, given a step identified with the GR with the initial contact time $t_{IC}$ and final contact time $t_{FC}$, we consider the XS signal $x_{train}$ corresponding to the adequate foot and define the pattern $p=x_{train}\left[t_{IC}:t_{FC}\right]$. This pattern *p* can be seen as a signal of length $N_{p}=t_{FC}-t_{IC}+1$ that represents the typical angular velocity of a foot during a step. The process is iterated for all the steps and for both feet: Each step identified with the GR forms a different pattern *p*. All patterns corresponding to the trial $i_{train}$ are stored in a library $P_{train}$.

### 3.2. Use of the Library to Detect the Steps

The library $P_{train}$ is used to detect the steps for the trial $i_{test}$ (which does not necessarily belong to the same subject and/or the same session). To that end, we consider the XS *Y*-axis angular velocity $x_{test}$ for trial $i_{test}$. Each pattern $p\in P_{train}$ is slid along signal $x_{test}$ and for each possible shift we compute the Pearson correlation coefficient. The final result is a matrix C of size $N_{P}\times N_{test}$, where $N_{P}$ is the number of templates in $P_{train}$ and $N_{test}$ is the number of samples of signal $x_{test}$, where

(1) $$\forall i_{p}\in 〚1,N_{P}〛,\;\forall i_{t}\in 〚1,N_{test}〛\;\;\;\;\;\;\;\;\;c\left(i_{p},i_{t}\right)=\mathrm{corr}\left(p,x_{test}\left[i_{t}:i_{t}+N_{p}-1\right]\right),$$

and corr(.,.) is the Pearson correlation coefficient.

The matrix C is then processed with an iterative and greedy detection strategy, described in [44], which detects steps by iteratively selecting the largest Pearson correlation coefficients in the matrix until all of them are lower than a threshold λ = 0.6. The influence of threshold λ is discussed in [44] and the value 0.6 insures that the algorithm does not consider irrelevant matches. The main idea behind this procedure is that we select the best possible templates in train trial $i_{train}$ to detect the steps in test trial $i_{test}$.

The output of the algorithm is a list of steps $S_{i_{test}|i_{train}}$ (steps of trial $i_{test}$ detected with the library of trial $i_{train}$). For each detected step $s\in S_{i_{test}|i_{train}}$, we also have access to the template $p_{s}\in P_{train}$ that was used for the detection, and to the Pearson correlation coefficient $c_{s}$ between *s* and $p_{s}$. Those additional outputs, which were not investigated in [44], are actually of interest since:

- Knowledge on $p_{s}$ allows to characterize the step *s*. Since we know that $p_{s}$ was the template in $P_{train}$ that most resembled step *s*, any available information on $p_{s}$ can be used to understand the shape, duration, length, etc., of step *s*. For instance, several steps detected with the same template are likely to be similar.
- Coefficient $c_{s}$ informs us of how strongly $p_{s}$ and *s* matched. A $c_{s}$ close to 1 implies that a pattern exists in $P_{train}$ that is very close to the phenomenon observed in step *s*; in other words, the confidence in step *s* is high. If, on the contrary, $c_{s}$ is small, no templates in $P_{train}$ exactly fitted the detected step *s*. This could mean that the locomotion of trial $i_{train}$ is different than the one of $i_{test}$.

### 3.3. Similarity Index

In order to use this additional information for the gait characterization, we propose to introduce a new parameter, called SId (similarity index). Given a library of template $P_{train}$ and a test trial $i_{test}$, this quantity is defined as

(2) $${\mathrm{SId}}_{i_{test}|i_{train}}=\underset{s\in S_{i_{test}|i_{train}}}{\mathrm{mean}}\left(c_{s}\right).$$

The SId is the mean of the Pearson correlation coefficients computed between detected steps and their respective closest templates. This quantity measures the ability of trial $i_{train}$ to detect the steps in trial $i_{test}$. It can be interpreted as a similarity index between trials $i_{train}$ and $i_{test}$ (assuming that if both trials were identical the step detection would be easy to perform and would produce large Pearson coefficients). It can also be seen as a confidence index on the detection (if this index is close to 1, it means that all detected steps were very similar to the annotated steps in the library and thus are likely to be well detected).

Note that the SId is not symmetrical, as using steps in trial $i_{train}$ to detect the steps in trial $i_{test}$ might not be the same as using steps in trial $i_{test}$ to detect the steps in trial $i_{train}$.

### 3.4. Use of the SId Index in Various Configurations

According to the chosen train and test sets, the SId index can be used either for longitudinal follow-up or inter-individual comparison. In this article, we consider four different configurations referred to as A1–A4.

- A1: Intra-individual intra-session. Two different trials belonging to the same subject and the same session (M0|M0 or M6|M6).
- A2: Intra-individual inter-session. Two trials belonging to the same subject but not to the same session (M0|M6 or M6|M0).
- A3: Inter-individual intra-group. Two trials belonging to different subjects of the same group (pMS|pMS or HS|HS).
- A4: Inter-individual inter-group. Two trials belonging to two subjects in different groups (pMS|HS or HS|pMS).

**Figure 3:**
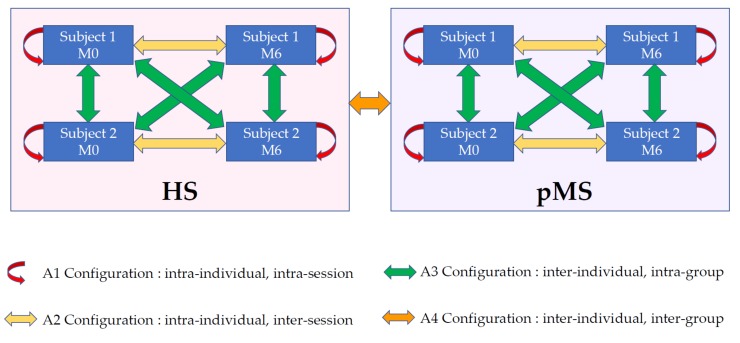
Definitions of the different pairs of extraction/detection trials that are analyzed in the article.

In order to investigate the properties of the SId index, we computed all SId between all trials of all subjects and merged the SId values according to these four different configurations, as illustrated in Figure 3.

### 3.5. Conventional Features

In addition to the SId, the following conventional gait parameters were computed:

- Average velocity: Velocity was computed for the way in (or the way out) as the total length of the detection part of the GR divided by the total time of the way in (or the way out). The average velocity was then the average of the velocity of the way in and the velocity of the way out.
- Step length: This parameter was extracted from the GR output.
- Step time: This parameter was computed as the average of the differences between the final contact time and the initial contact times given by the GR.
- Double stance time: This parameter was computed as the average of the differences between the final contact time of one foot and the initial contact times of the contralateral foot given by the GR.
- Variation coefficient of step time: This parameter was computed as the standard deviation of the differences between the final contact time and the initial contact times given by the GR divided by the step time.
- Variation coefficient of double stance time: This parameter was computed as the standard deviation of the differences between the final contact time of one foot and the initial contact times of the contralateral foot given by the GR divided by the step time.

Given two gait trials, we computed the differences between the parameter values and merged these differences with the four different configurations illustrated in Figure 3.

### 3.6. Link to the Performance of the Step Detection

The similarity index (SId) can be interpreted as a confidence index for the step-detection algorithm. Indeed, a large SId suggests that the patterns present in the train library fit those observed in the test signal and are therefore likely to provide efficient detection. To investigate this question, we computed the correlation between the SId values and some evaluation metrics commonly used for assessing the performances of step-detection algorithms [44]. These metrics are based on the ground truth step annotations provided by the GR.

- **Precision (or positive predictive value).** A detected step is counted as correct if the mean of its start and end times lies inside an annotated step. An annotated step can only be detected one time. If several detected steps correspond to the same annotated step, all but one are considered as false. The precision is the number of correctly detected steps divided by the total number of detected steps.
- **Recall (or sensitivity).** An annotated step is counted as detected if the mean of its start and end times lies inside a detected step. A detected step can only be used to detect one annotated step. If several annotated steps are detected with the same detected step, all but one are considered undetected. The recall is the number of detected annotated steps divided by the total number of annotated steps.
- **F-measure (or F1 score)**. The F-measure is the harmonic mean of precision and recall.
- **ΔStart.** For a correctly detected step, this is the difference between the detected start time and the annotated start time.
- **ΔEnd.** For a correctly detected step, this is the difference between the detected end time and the annotated end time.
- **ΔDuration.** For a correctly detected step, this is the difference between the duration of the detected step and the duration of the annotated step.

### 3.7. Statistics

All parameters were tested for normality using Shapiro-Wilks tests. Parametric tests were applied for normal distributions and non-parametric tests were resorted to when this hypothesis was rejected. Means and standard deviations (SD) were reported, except for ordinal distributions (EDSS) where mean and interquartile range were reported.

#### 3.7.1. Comparisons between Configurations

SId and change in gait conventional features were compared between configurations of pairs of extraction/detection trials using the absolute difference between the mean value in the two groups. For all these non-parametric variables, the Krustkall-Wallis test—a rank-based non-parametric test used to assess more than two independent groups—was used. Rejection of the null-hypothesis was followed by subsequent Wilcoxon tests to test differences in medians. All tests were corrected for multiple comparisons using Bonferroni adjustment. For each group (HS and pMS, respectively), the percentile score of SId from A2 was computed from the distribution of SId from A3. The percentile score of SId from A2 was also computed from the distribution of SId from A4.

#### 3.7.2. Correlations

SId was correlated to performance, accuracy and conventional gait features using Pearson moment product correlation coefficients, which remains a valid method, even in the case of non-normal datasets [45]. Pearson correlation coefficient is interpreted as very high for absolute values between 0.9 and 1.0, high for absolute values between 0.7 and 0.9, moderate for absolute values from 0.5 to 0.7, low for absolute values from 0.3 to 0.5 and negligible for absolute values below 0.3 [46].

Primary data analysis (extraction and detection process) was done using MATLAB^®^ R2019a. Statistical analysis was performed using R v3.5.1. All tests were corrected for multiple comparisons using Bonferroni adjustment.

## 4. Results

In this section, we investigate the ability of this index to effectively compare two trials. To investigate the potential of SId as a gait biomarker, three different and complementary questions are investigated:

- Comparison of SId based on these four configurations: Comparing SIds computed within the same session (A1), SIds computed from different sessions of the same subject (A2), SIds computed between subjects of the same group (A3) and SIds computed between groups (A4).
- Correlation of SId with more conventional features used to characterize gait (average velocity, step length, step time, double stance time, variation coefficient of step time, variation coefficient of double stance time).
- Correlation of SId with the detection performance of the step-detection algorithm.

### 4.1. Comparison of SId Based on Configurations

**Figure 4:**
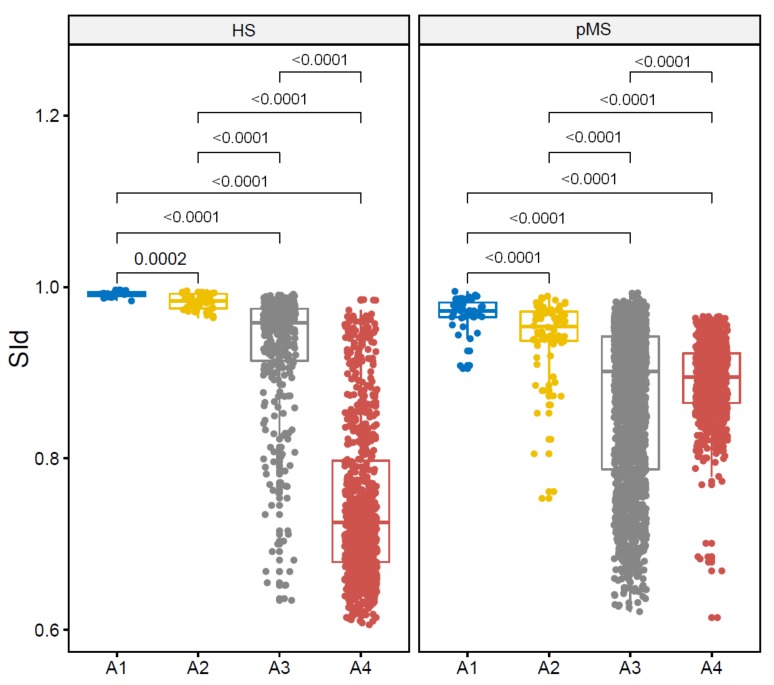
Comparison of SId predictions across configurations: Intra-individual intra-session prediction (A1) vs. intra-individual inter-session prediction (A2) vs. intra-group inter-individual prediction (A3) vs. inter-group inter-individual prediction (A4).

In this experiment, the values of SId are compared between the four configurations: Intra-individual intra-session comparison (A1), intra-individual inter-session comparison (A2), intra-group inter-individudal comparison (A3) and inter-group inter-individudal comparison (A4). Boxplots are displayed in Figure 4. SId shows its highest values for the A1 comparison (HS: 0.99 (0.00), pMS: 0.97 (0.01)) and decreases from A1 to A4, both in the HS and the pMS group (*p*-value of the Krustkall-Wallis test: <0.0001, *p*-value of the subsequent Wilcoxon tests: <0.0001 for all paired comparisons). In particular, it shows that trials from a given subject are closer to each other than to trials from another subject both in the HS group (mean difference: 0.046; *p*-value: <0.0001) and in the pMS group (mean difference: 0.055; *p*-value: <0.0001). Comparisons of A3 (inter-individual intra-group) and A4 (inter-individual inter-group) show that SIds obtained for intra-group comparison are larger than inter-group ones in the HS group (mean difference: 0.190; *p*-value: <0.0001) but not in the pMS group (mean difference: 0.070; *p*-value = 0.52).

For a given individual *k* inside the HS or the pMS groups, SIds for comparison of one trial to another trial are reproduced in Table 2. This table shows that, on average, trials from a given subject are closer to other trials from the same subject than to trials from other subjects. For HS subjects, SId from A2 prediction belongs to the 90th (SD: 14) percentile of the distribution of SId from A3 prediction and is always higher than SId from A4. For pMS subjects, SId from A2 prediction belongs to the 96th (SD: 8) percentile of the distribution of SId from A3 prediction and the 99th (SD: 2) percentile of the distribution of SId from A4.

### 4.2. Correlation of SId with Conventional Features

**Figure 5:**
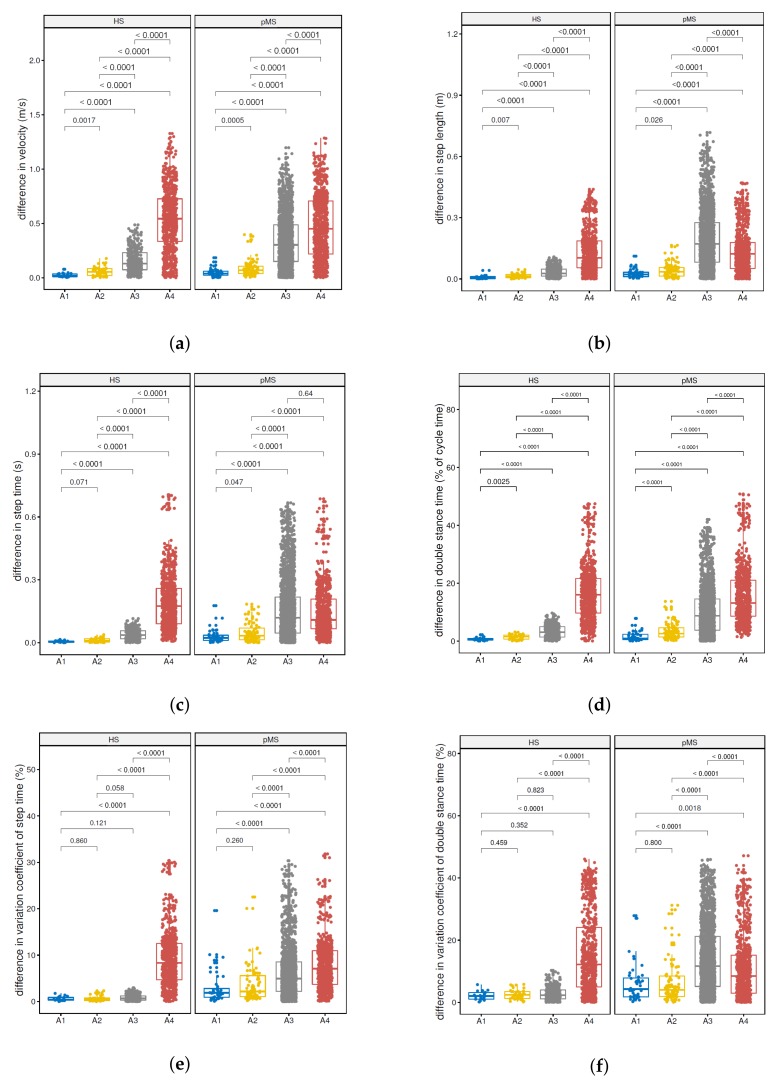
Intra-individual intra-session prediction (A1) vs. intra-individual inter-session prediction (A2) vs. intra-group inter-individual prediction (A3) vs. inter-group inter-individual prediction (A4) for both cohorts: (**a**) Average walking velocity; (**b**) step time; (**c**) step length; (**d**) double stance time; (**e**) coefficient of variation of step time; (**f**) coefficient of variation of double stance time.

Comparisons were also carried out for the average walking velocity (Figure 5a), step length (Figure 5b), step time (Figure 5c), double stance time (Figure 5d), coefficient of variation of step time (Figure 5e) and coefficient of variation of double stance time (Figure 5f), which are classical gait features [4]. After controlling for multiple comparisons, difference in average velocity (Figure 5a) and differences in double stance time (Figure 5d) proved significantly higher in the A2 (intra-individual inter-session) comparison as compared to the A1 (intra-individual intra-session) comparison in the HS group (*p*-values of 0.002 and 0.003, respectively, with a threshold of 0.017) and the pMS group (*p*-values < 0.001 and < 0.0001, respectively, with a threshold of 0.017). Difference in step length (Figure 5b) was also higher in the A2 comparison as compared to the A1 comparison in the HS group (*p*-values of 0.007, with a threshold of 0.017). All other comparisons of configurations were highly significant (*p*-value < 0.0001).

**Figure 6:**
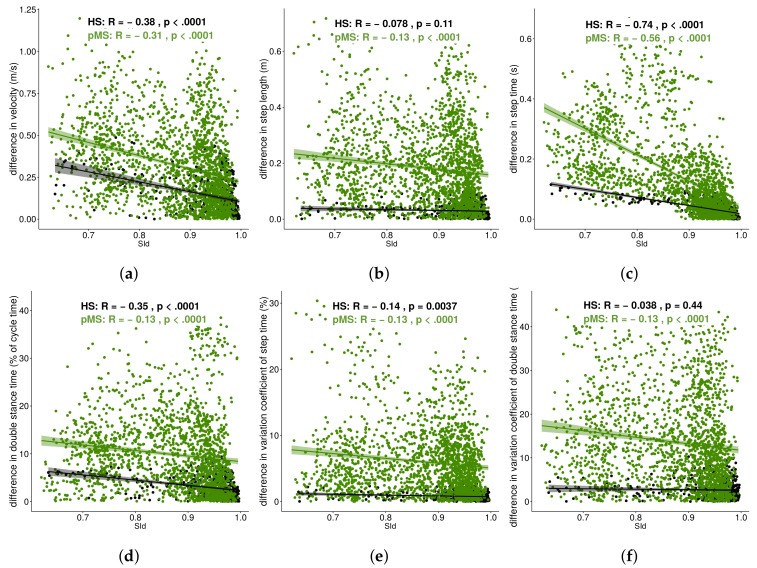
Correlation of SId to conventional features: (**a**) Average walking velocity; (**b**) step length; (**c**) step time; (**d**) double stance; (**e**) coefficient of variation of step time; (**f**) coefficient of variation of double stance time.

To investigate how SId would correlate to change in these conventional features, SId, as measured for each intra-group comparison (A1, A2, A3), was correlated to variation between the respective train trial and test trial for each of the following conventional gait features: The average walking velocity (Figure 6a), step time (Figure 6b), step length (Figure 6c), double stance time (Figure 6d), coefficient of variation of step time (Figure 6e) and coefficient of variation of double stance time (Figure 6f).

For both groups, low correlations were observed for difference in the average walking velocity (Figure 6a) (HS: r=-0.38, *p*-value: < 0.0001; pMS: r=-0.31, *p*-value: < 0.0001), double stance time (Figure 6d) (HS: r=-0.35, *p*-value: < 0.0001; pMS: r=-0.13, *p*-value: < 0.0001) and the variation coefficient of step time (Figure 6e) (HS: r=-0.14, *p*-value: 0.004; pMS: r=-0.13, *p*-value: < 0.0001). Moderate to high correlations were observed for difference in step time (Figure 6c) (HS: r=-0.74, *p*-value: < 0.0001; pMS: r=-0.56, *p*-value: < 0.0001). Additional low correlation was seen for pMS participants for the difference in step length (Figure 6b) (r=-0.13, *p*-value: < 0.0001) and the variation coefficient of double stance time (Figure 6f) (r=-0.13, *p*-value: < 0.0001).

### 4.3. Correlation to Performance of the Step Detection

Performance and accuracy scores, along with their correlations to SId, are reported in Table 3. In the HS group, SId correlates moderately to the F-measure, ΔStart and ΔDuration, and weakly to ΔEnd. In the pMS group, SId correlates moderately to the F-measure and strongly to ΔStart and ΔDuration, while a very low correlation is found with ΔEnd.

## 5. Discussion

This study shows that SId is a valid metric to compare two gait trials both between different subjects or between two visits of a same subject to track changes in gait. In addition, in our small sample of patients, SId seems to give an insight into the performance of the underlying template-based step-detection method.

First, SId showed decreasing values from intra-individual intra-session (A1) to intra-individual inter-session (A2) to intra-group inter-individual (A3) to inter-group inter-individual (A4) trial comparisons for both the HS group and the pMS group. The difference in SId between A1 and A2 was expected for pMS individuals, for which symptoms vary from day to day depending, for instance, on the level of exercise and physical therapy or the weather (Uhtoff effect [47,48,49]). This higher change in HS participants between trials of different sessions compared to between trials of a same session was also true for conventional features. Average velocity and double stance time, as well as step length in the HS group, both displayed a higher difference when comparing inter-session with intra-session trials. Still, for all features, the difference in A2 remains within the standard error mean for inter-session comparison as found in the literature [50,51,52]. Furthermore, the hierarchy of variability in gait parameters is also found in the literature in intra-class correlation coefficients for both healthy subjects [19,53] and mixed groups of patients and subjects [10].What is more, SId shows high variability in between-cohort comparisons as compared to intra-cohort comparison for the HS group but not for the pMS group. Two participants from the pMS cohort can then be as distant as one participant from the pMS cohort and one participant from the HS cohort. One explanation is that pMS patients present with a wide range of gait alterations both in terms of the types of symptoms (which can relate to balance deficit, spasticity, decreased muscular strength, etc.) and severity of symptoms. In that regard, it can be observed in Figure 4 and Table 2 that the SId for the detection of steps from HS individuals using steps from pMS individuals seems lower than the detection of steps from pMS individuals using steps from HS individuals, which illustrates the non-symmetrical characteristic of the SId. This difference may be due to the durations of the steps that are different for HS and pMS subjects [23,54,55]. Due the greedy aspect of the matching procedure, it is easier for the algorithm to detect one large step with several small steps than the opposite. Therefore, higher SId values can be achieved by using HS templates to detect pMS steps than the opposite. One other explanation might that the noise level is larger for pMS subjects, thus creating noisy templates that are more difficult to match than HS smoother templates.

Second, as mentioned above, lower SId was associated with increased difference in step time between the train and test trial, a parameter which also showed strong correlation with disease severity as measured using the Expanded Disease Status Scale [16,23,54,55,56]. The SId has, therefore, potential to give insight into the evolution of the disease, without needing any pre-processing and step detection. However, even though most of them were significant, only low correlations were found for the differences in other conventional features that are usually used to characterize gait. As a matter of fact, very high variability in the difference of conventional features is seen, and one ought to be careful in drawing conclusions before larger and longer studies are carried out.

Third, SId has been shown to provide key information on the underlying step-detection algorithm. One major drawback of automatic step-detection algorithms is that it is tricky to assess their performances in real-life conditions. In particular, when confronted with different types of gait or cohorts, their accuracy may drop, which can have consequences if they are used in a clinical context. As a matter of fact, most of the algorithms designed for a particular type of subject may suffer from degraded performance in other cohorts [57]. Thanks to its construction, a large SId between two trials means that templates used to detect the steps were close to these latter. Very low SId values can therefore be interpreted as a discrepancy between the train and test trial, which is likely to cause a poor step detection. Indeed, we showed in Table 3 a correlation between SId and performance as well as accuracy scores. The SId values are therefore linked to the confidence in the underlying detection algorithm, and could be used to report that the model used in the detection process does not suit the tested data. If several libraries of templates were available (e.g., one for each cohort or one for each gait disability), the SIds could be used to select the most appropriate library and thus improve the step-detection performances. These perspectives shall be investigated in future studies.

Eventually, these results can be applied to a wide range of pMS individuals, with mild as well as severe diseases. Indeed, as patients using walking aids were also included, the conclusions also apply to patients with EDSS 6 and 6.5, which fills a gap in the literature [23]. Comparisons of SId between other populations should be informative to compare distances between gaits of patients disabled by different Neurological illness and participates in the development of a new taxonomy. New matching procedures may also be implemented, for instance, by using Dynamic-Time Warping (DTW), which allows to match time series of different lengths. In particular, the use of this technique dedicated to template matching may be useful in the context of step detection and recognition [58].

Our study has limitations. First, sampling fluctuations may have occurred due to the small sample size, particularly of HS. Recruiting young healthy subjects was difficult due to the necessity of a six month time period between both measurements. In particular, strong variability was found when correlating conventional features with the SId. Even though results were significant, the clouds of points are sparse.

## 6. Conclusions

In this article, we introduced a novel algorithm for comparing inertial signals of two gait trials. The output parameter, a metric referred to as the similarity index (SId), is comprised between 0 and 1 and reflects how similar two gait trials are. This parameter shows promising results for the longitudinal follow-up of participants, as it is sensitive to changes in gait features. Larger studies are needed to confirm the potential of SId as a predictor of changes and a longer follow-up time could also allow assessment of its prognostic value. Besides, as the SId correlates to the performance and accuracy of the underlying step-detection algorithm, it provides immediate feedback of the detection, which is a key aid for decision making.

## Figures and Tables

**Table 1:** Baseline characteristics of patients with progressive multiple sclerosis (pMS) and healthy subjects (HS). For the age, height, weight, BMI, Multiple Sclerosis Walking Scale (MSWS) and Fatigue Impact Scale (FIS), the mean and the standard deviation (SD) are displayed. For the Expanded Diseases Status Scale (EDSS) and the functional scores (subscores of EDSS), the statistics are reported as median and interval quartile range (IQR).

	pMS (*n* = 22)	HS (*n* = 10)
**Sex (M/F)**	9/13	4/6
**Age (years)**	58 (11)	26 (1)
**Height (m)**	1.71 (0.09)	1.72 (0.09)
**Weight (kg)**	71.2 (16.6)	58.2 (10.9)
**BMI (kg/m${}^{2}$)**	24.3 (5.1)	21.0 (3.0)
**EDSS**	5.0 [3.5–6]	-
EDSS—pyramidal	3.0 [3.0–3.8]	-
EDSS—cerebellar	1.5 [0.0–3.0]	-
EDSS—bulbar	0 [0.0–0.8]	-
EDSS—sensitive	2.0 [1.0–2.0]	-
EDSS—cognitive	1.0 [0.0–2.0]	-
**MSWS**	65.0 (17.3)	-
**FIS**	43.4 (24.9)	-
**Use of walking aid for the walk test (/total number)**	7/22	0/10
Cane (1 or 2)	4	-
Walker	1	-
Human help	1	-
Cane + human help	1	-

**Table 2:** Similarity index scores for comparing one gait trial depending on the training trial (intra-individual inter-session, intra-group inter-individual, inter-group inter-individual). Means and standard deviations are displayed for both pMS and HS groups.

	HS		pMS	
	Individual k	Other Individual	Individual k	Other Individual
**HS (individual k)**	0.98 (0.01)	0.93 (0.07)	-	0.75 (0.09)
**pMS (individual k)**	-	0.89 (0.04)	0.94 (0.05)	0.87 (0.09)

**Table 3:** Correlations between SId and the F-measure and accuracy scores for the step detected. All configurations are pooled together and reported as mean (SD).

	HS (n=10)			pMS (n=22)		
	Value	Pearson	*p*-Value	Value	Pearson	*p*-Value
F-measure	0.843 (0.213)	0.560	<0.0001	0.934 (0.130)	0.548	<0.0001
ΔStart	0.18 (0.164)	−0.580	<0.0001	0.154 (0.170)	−0.781	<0.0001
ΔEnd	0.066 (0.087)	−0.306	<0.0001	0.026 (0.035)	−0.084	0.0001
ΔDuration	0.234 (0.209)	−0.548	<0.0001	0.173 (0.179)	−0.771	<0.0001
